# Differential evolution with classified mutation for parameter extraction of photovoltaic models

**DOI:** 10.1371/journal.pone.0332083

**Published:** 2025-10-09

**Authors:** Yong-Gang Chen, Yan Cao, Kai Lu, Quanxin Yang, Yange Chen, Yuan Ping

**Affiliations:** 1 School of Information Engineering, Xuchang University, Xuchang, China; 2 Henan Province Engineering Technology Research Center of Big Data Security and Application, Xuchang, China; 3 Henan International Joint Laboratory of Polarization Sensing and Intelligent Signal Processing, Xuchang, China; UCSI University, MALAYSIA

## Abstract

Existing algorithms for photovoltaic (PV) parameter extraction struggle to balance accuracy and computational efficiency when handling complex models. To address this gap, a differential evolution with classified mutation (DECM) is proposed, which integrates adaptive mutation strategies and a hierarchical classification framework to improve both scalability and precision. In DECM, all individuals are divided into many subswarms. The best position in each sub-swarm is considered the locally best position. Two different mutation strategies are developed for these local best positions. For the other individual positions, a different mutation strategy is used to improve these positions. The DECM utilizes a multi-swarm approach to allocate specific roles to individual particles, followed by the implementation of role-specific mutation strategies. In contrast to some other differential evolution algorithms, the DECM eliminates both crossover operations and parameter tuning strategies, thereby offering enhanced simplicity and operational efficiency. To better understand the effectiveness of DECM, several photovoltaic models are adopted. According to the experimental results, DECM outperforms some popular algorithms in terms of solution accuracy, computational efficiency and parameter extraction robustness.

## 1 Introduction

Energy is the basis for human survival and sustainable development. Today there are many problems in the world such as inadequate energy supply, environmental pollution and rapid global warming, and these problems are becoming more and more serious. Resolving the contradiction between energy supply and demand is an urgent problem. Currently, fossil fuels dominate energy consumption and production. The use of fossil fuels can cause serious pollution problems. In order to achieve sustainable development, humanity is committed to the development of renewable energy. Obviously, as a renewable energy, solar energy is an inexhaustible and ideal energy for billions of years [1]. A photovoltaic (PV) system can convert solar energy into electrical energy. As part of the global trend towards reducing CO_2_ emissions, the share of photovoltaic power generation is increasing in more developed regions. People have designed different mathematical models for different photovoltaic systems. An accurate photovoltaic model can improve the performance of a photovoltaic system. The parameters of the photovoltaic model have an important influence on the accuracy of the photovoltaic model. Therefore, accurate extraction of parameters is a valuable research direction. So far, researchers have proposed some parameter extraction methods. Among these methods, analytical methods and numerical methods are the two main methods. Deterministic methods and metaheuristic methods are numerical methods. Metaheuristic methods have many advantages over other methods [2]. Therefore, researchers have developed more and more metaheuristic algorithms to solve the problem of parameter extraction in PV models.

In [3], a multitask evolutionary optimization algorithm (SGDE) was used to obtain the parameter values of three PV models. SGDE combines the similarity measurement method, a differential evolutionary (DE) mutation strategy and the chaotic local search method of the elite mechanism. Liu et al. [4] developed an advanced slime mold algorithm (CNMSMA) to extract the parameters. The results confirmed that CNMSMA had accuracy and satisfactory stability. An enhanced JAYA optimization algorithm (EJAYA) was proposed in [5]. The EJAYA algorithm incorporates an adaptive evolution operator, a population resizing strategy, and an opposition-based learning mechanism. Experimental results demonstrate its exceptional performance under varying temperature and irradiance conditions. In [6], FC-EPSO was proposed. The fractional chaos maps was used to improve the accuracy and reliability in FC-EPSO. The statistical measures and comparative studies showed the effectiveness of FC-EPSO. Lin and Wu designed a niche particle swarm optimization (NPSOPC) [7]. Jing et al. developed CPMPSO to get relevant parameters values [8]. In CPMPSO, based on the evaluation results, different personal best positions adopt different differential mutation strategies. Experiments confirmed the effectiveness of CPMPSO. In [9], the proposed Enhanced Prairie Dog Optimizer (En-PDO) establishes a new benchmark in photovoltaic parameter estimation, offering enhanced precision and robustness across diverse solar cell models through its innovative integration of random learning and logarithmic spiral search strategies. In [10], the proposed NSGNDO algorithm establishes itself as a robust solution for photovoltaic system parameter estimation, achieving unprecedented precision (RMSE of 9.8248E-04 for RTC France solar cell) and outperforming competing methods through its innovative integration of neighborhood search strategies with normal distribution optimization. Yan et al. [11] developed another brainstorming optimization algorithm (IBSO). In the IBSO, a scheme for generating new individuals and a scheme for updating self-adaptive individuals were introduced. In addition, an improved clustering scheme has been developed in IBSO. Experimental results suggest that IBSO was competitive. Abdelkader et al. developed an opposition-based learning modified Salp swarm algorithm (OLMSSA) [12]. A new algorithm (IGSK) was introduced by Karam et al [13]. Compared to classic knowledge acquisition and knowledge exchange, IGSK has added three mechanisms. Based on these three improvements, IGSK achieved better performance compared to other comparison algorithms. In [14], an enhanced bat algorithm based on L$\overset{´}{e}$vy flight (ELBA) was used to find the different parameters of PV modules. Compared to the bat algorithm, ELBA mainly introduces three innovations. In [15], QIOA is proposed to identify the parameters. The proposed QIOA approach, incorporating VVR-based modeling, establishes a new benchmark in PV parameter extraction, outperforming conventional algorithms in accuracy, convergence speed, and robustness for both single-crystalline and multi-crystalline silicon cells under varying radiation conditions. A LaPSO was proposed in [16]. LaPSO employed a landscape-based adaptive operator selection strategy to identify the parameters. There are more and more results of parameter extraction using differential evolution (DE) [17–25]. Despite the numerous studies on differential evolution algorithms for parameter extraction of photovoltaic models, most existing algorithms still rely on crossover operations and parameter tuning strategies in the optimization process. This not only increases the complexity of the algorithm but also leads to reduced computational efficiency. Moreover, existing algorithms often fail to balance accuracy, robustness, and convergence speed when dealing with different photovoltaic models. Therefore, developing a differential evolution algorithm that is both simple and efficient is of great significance for parameter extraction of photovoltaic models.

Due to the complexity and diversity of the PV mathematical model, it is a challenging problem to accurately determine the parameter values. These methods presented above have made great progress in parameter extraction. However, according to the experimental results, some methods are not ideal for some PV models. Moreover, existing algorithms often fail to balance accuracy, robustness, and convergence speed when dealing with different photovoltaic models. Therefore, it is necessary to design some metaheuristic algorithms with better performance for parameter extraction. After analyzing some literature, it can be found that various differential mutation strategies play an important role in parameter extraction in [8,11,17,26]. In addition, some differential evolution algorithms have good performance in PV parameter extraction. The multi-swarm technique has attracted increasing attention. Since multi-swarm technology has some advantages, it is widely used in some swarm intelligence algorithms [27–32]. The fitness-based multirole and ensemble mutation strategies are widely used to improve the performance of some algorithms [33–36]. Based on the above thoughts and observations, the multi-swarm strategy and the role-based classification mutation strategy are adopted in DECM. Considering that different mutation strategies have different properties. It makes sense to design different mutation strategies according to individual roles. According to the multi-swarm structure of DECM, two mutation strategies are designed for the best individual of each subswarm. A different mutation strategy is used to evolve other individuals. Several standard PV models are used to test the performance of DECM. The experimental results fully demonstrate that the DECM algorithm outperforms the comparison algorithm in terms of comprehensive indicators.

Here are the main points and highlights of this paper:

(1) A novel variant of differential evolution called DECM is proposed. According to the characteristics of a multi-swarm, a better combination of different mutation strategies is found for different individuals. The DECM algorithm distinguishes itself from other differential evolution algorithms by eschewing crossover operations and parameter adaptation, instead relying solely on the optimization of three differential mutations. This streamlined approach confers the benefits of simplicity in implementation, clarity in comprehension, and expediency in computational performance.

(2) The detailed analysis and comparative study for parameter extraction was carried out. Experimental results demonstrate that DECM achieves lower RMSE values across multiple PV models compared to other popular algorithms, highlighting its superior accuracy and robustness. DECM significantly reduces running time without sacrificing parameter extraction accuracy, making it more suitable for practical applications in PV systems. DECM shows strong adaptability in different environmental conditions, such as varying irradiance and temperature, proving its practical value in renewable energy systems.

(3) Based on experimental results, some useful conclusions can be drawn. It turns out that it is necessary to develop new mutation strategies. Combining different mutation strategies is effective, and exploring different combinations of mutation strategies is a worthwhile direction. Some parameter adjustment strategies can extend the CPU running time.

(4) Some new perspectives have been opened for the design of algorithms with high precision, high speed and high efficiency. Some possible research directions are highlighted.

Below are the sections of this document. In Sect 2, several PV models and associated formulas of these models are briefly introduced. In Sect 3, the basic DE is presented. A detailed description of the proposed DECM can be found in Sect 4. Sect 5 presents the experimental results and analysis of the results. Finally, Sect 6 summarizes the results of this research.

## 2 PV models and its formulation

**Fig 1:**
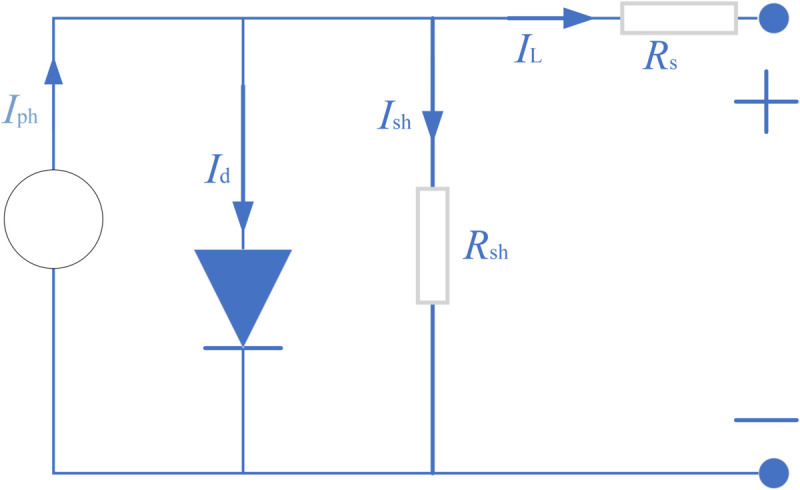
Equivalent circuit of SDM.

**Fig 2:**
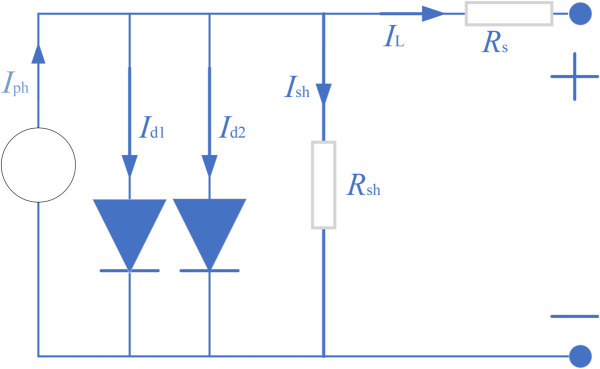
Equivalent circuit of DDM.

**Fig 3:**
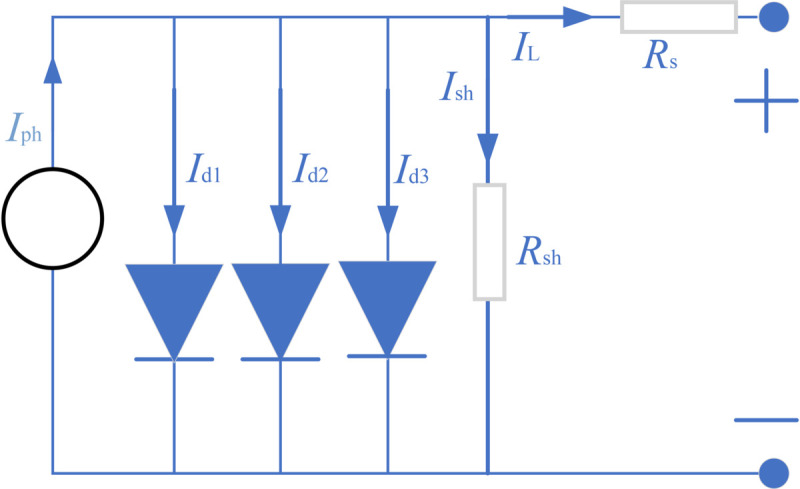
Equivalent circuit of TDM.

**Fig 4:**
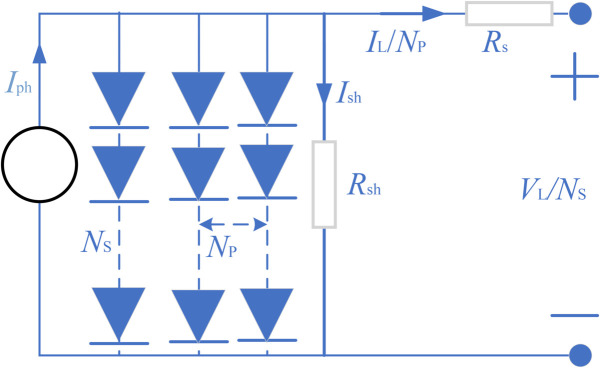
Equivalent circuit of the PV model.

As described in the mathematical models for these PV systems, the most popular models are the single diode model (SDM), the double diode model (DDM), the three-diode model(TDM) and the PV module model (PVMM). Below is a brief description of each model and its objective function. Fig 1 presents the equivalent circuit for SDM of a photovoltaic (PV) system. This model simplifies the solar cell’s electrical behavior using one diode. Fig 2 displays the equivalent circuit for DDM. This model improves upon the SDM by adding a second diode to better capture the cell’s recombination currents. Fig 3 outlines the equivalent circuit for TDM. With three diodes representing distinct recombination processes, this model delivers an even more detailed simulation of solar cell behavior. Fig 4 shows the equivalent circuit for PVMM. This model represents a full PV module with multiple series and parallel cell connections.

According to these figures, the output current ($I_{L}$) expression formulas of these models are Eqs 1–4, respectively. Table 1 lists the string symbols and their representative meaning. Detailed information about these models can be found in references [37–40]

**Table 1:** String symbols and meanings.

string symbols	meanings	string symbols	meanings
*I*_*ph*_	photo-generated current(A)	*I*_*d*_,*I*_*d*1_,*I*_*d*2_,*I*_*d*3_	diode currents(*μ*A)
*I*_*sd*_	reverse saturation current of diode	*I*_*sh*_	shunt resistor current(A)
*I*_*sd*1_	diffusion current	*I*_*sd*2_	saturation current(A)
*I*_*sd*3_	leakage current	*R*_*s*_	series resistance
*n*,*n*_1_,*n*_2_,*n*_3_	diode ideal factors	*T*	cell temperature(k)
*k*	Boltzmann constant (1.3806503*10^−23^)	*q*	electron charge(1.60217646*10^−19^)
$V_{L}$	terminal voltage	*R*_*sh*_	parallel resistance
*N*_*p*_	the number of diodes in parallel	*N*_*s*_	the number of diodes in series
*N*	the number of experimental data	RMSE	root mean square error

$$I_{L}=I_{ph}-I_{sd}*[exp(\frac{q*(V_{L}+R_{s}*I_{L})}{n*k*T})-1]-\frac{V_{L}+R_{s}*I_{L}}{R_{sh}}$$ (1)

$$I_{L}=I_{ph}-I_{sd1}*[exp(\frac{q*(V_{L}+R_{s}*I_{L})}{n_{1}*k*T})-1]\;\;-I_{sd2}*[exp(\frac{q*(V_{L}+R_{s}*I_{L})}{n_{2}*k*T})-1]-\frac{V_{L}+R_{s}*I_{L}}{R_{sh}}$$ (2)

$$I_{L}=I_{ph}-I_{sd1}*[exp(\frac{q*(V_{L}+R_{s}*I_{L})}{n_{1}*k*T})-1]-I_{sd2}*[exp(\frac{q*(V_{L}+R_{s}*I_{L})}{n_{2}*k*T})-1]\;\;-I_{sd3}*[exp(\frac{q*(V_{L}+R_{s}*I_{L})}{n_{3}*k*T})-1]-\frac{V_{L}+R_{s}*I_{L}}{R_{sh}}v$$ (3)

$$I_{L}/N_{p}=I_{ph}-I_{sd}*[exp(\frac{q*(V_{L}/N_{s}+R_{s}*I_{L}/N_{p})}{n*k*T})-1]\;\;-\frac{V_{L}/N_{s}+R_{s}*I_{L}/N_{p}}{R_{sh}}$$ (4)

### 2.1 Objective function

Obviously, the key of solving parameter extraction is the definition of optimization objective functions. This work aims at minimizing the error between measured and simulated current.

The current error functions *f*($V_{L}$,*I*_*L*_, *x*) of the three models are formulated in Table 2, where the vector *x* represents the aggregate of unknown parameters to be extracted in these models.

**Table 2:** The formulas of different PV models.

Model	Formulas	unknown parameters
SDM	$f(V_{L},I_{L},x)=I_{ph}-I_{sd}*[exp(\frac{q*(V_{L}+R_{s}*I_{L})}{n*k*T})-1]-\frac{V+R_{s}*I_{L}}{R_{p}}-I_{L}$	$I_{ph},I_{sd},R_{s},R_{sh},n$
	$x=\{\begin{array}{c} I_{ph},I_{sd},R_{s},R_{sh},n \end{array}\}$	
DDM	$f(V_{L},I_{L},x)=I_{ph}-I_{sd1}*[exp(\frac{q*(V_{L}+R_{s}*I_{L})}{n_{1}*k*T})-1]-$	$I_{ph},I_{sd1},I_{sd2},$
	$I_{sd2}*[exp(\frac{q*(V_{L}+R_{s}*I_{L})}{n_{2}*k*T})-1]-\frac{V_{L}+R_{s}*I_{L}}{R_{sh}}-I_{L}$	$R_{s},R_{sh},n_{1},n_{2}$
	$x=\{\begin{array}{c} I_{ph},I_{sd1},I_{sd2},R_{s},R_{sh},n_{1},n_{2} \end{array}\}$	
TDM	$f(V_{L},I_{L},x)=I_{ph}-I_{sd1}*[exp(\frac{q*(V_{L}+R_{s}*I_{L})}{n_{1}*k*T})-1]-I_{sd2}$	$I_{ph},I_{sd1},I_{sd2},I_{sd3},$
	$*[exp(\frac{q*(V_{L}+R_{s}*I_{L})}{n_{2}*k*T})-1]-I_{sd3}*[exp(\frac{q*(V_{L}+R_{s}*I_{L})}{n_{3}*k*T})-1]-\frac{V_{L}+R_{s}*I_{L}}{R_{sh}}-I_{L}$	$R_{s},R_{sh},n_{1},n_{2},n_{3}$
	$x=\{\begin{array}{c} I_{ph},I_{sd1},I_{sd2},I_{sd3},R_{s},R_{sh},n_{1},n_{2},n_{3} \end{array}\}$	
PVMM	$f(V_{L},I_{L},x)=I_{ph}N_{p}-I_{sd}N_{p}*[exp(\frac{q*(V_{L}/N_{s}+R_{s}*I_{L}/N_{p})}{n*k*T})-1]-$	$I_{ph},I_{sd},R_{s},R_{sh},n$
	$\frac{N_{p}*V_{L}/N_{s}+R_{s}*I_{L}}{R_{sh}}-I_{L}$	
	$x=\{\begin{array}{c} I_{ph},I_{sd},R_{s},R_{sh},n \end{array}\}$	

As shown in Eq 5, RMSE can be considered as the objective function. The smaller the value of RMSE, the more accurate the parameter extraction is.

$$\text{min}\;\text{RMSE}=\sqrt{\frac{1}{N}\sum_{k=1}^{N}f(V_{L},I_{L},x{)}^{2}}$$ (5)

## 3 Differential evolution

As one kind of widely used optimization algorithm, DE was proposed in 1997 [41]. In DE, the population of a *D*-dimensional hyperspace is random at the beginning. The individuals of the *k*th generation can be represented by $X_{i}^{k}$=($x_{i,1},x_{i,2},...,x_{i,D}$), where k=0,1,...,G denotes the generation time and G is maximal times of the generation, where i=1,2,...,NP denotes the *i*th individual and the total number of individuals is called *NP*. In DE, the evolution of each individual in each generation mainly depends on three operations: crossover, mutation and selection.

### 3.1 Mutation

Mutation is a change or disturbance to individuals. at the *k* generation, DE utilized some individuals $X_{i}^{k}$ to obtain the mutant vector $V_{i}^{k}$. Based on some literatures [20,31], there are several commonly mutation strategies as follows:

DE/rand/1

$$V_{i}^{k}=X_{r1}^{k}+F\cdot (X_{r2}^{k}-X_{r3}^{k})$$ (6)

DE/current-to-best/1

$$V_{i}^{k}=X_{i}^{k}+F\cdot (X_{best}^{k}-X_{i}^{k})+F\cdot (X_{r1}^{k}-X_{r2}^{k})$$ (7)

DE/best/1

$$V_{i}^{k}=X_{best}^{k}+F\cdot (X_{r1}^{k}-X_{r2}^{k})$$ (8)

DE/best/2

$$V_{i}^{k}=X_{best}^{k}+F\cdot (X_{r1}^{k}-X_{r2}^{k})+F\cdot (X_{r3}^{k}-X_{r4}^{k})$$ (9)

DE/rand/2

$$V_{i}^{k}=X_{r1}^{k}+F\cdot (X_{r2}^{k}-X_{r3}^{k})+F\cdot (X_{r4}^{k}-X_{r5}^{k})$$ (10)

The subscripts *r*1, *r*2, *r*3, *r*4, *r*5 are mutually distinct integers and unequal to the index *i*. These subscripts are randomly produced from 1,2,...,*NP*. *F* is the scaling factor of difference vector. $X_{best}^{k}$ denotes the best individual, which is a vector with the best fitness value in the current population. Note that some dimensions of the trial vector $V_{i}^{k}$ may violate search boundary constraints. In this case, the boundary handling technique can be expressed as follows:

$$V_{i,d}^{k}=LB_{d}+randv\cdot (UB_{d}-LB_{d})$$ (11)

Where d∈[1,D], *randv* is a random number in (0,1), and *LB*_*d*_, *UB*_*d*_ are the lower and upper bound in the *d*th dimensions, respectively.

### 3.2 Crossover

The crossover operation can generate the crossover vector (trial vector) $u_{i}^{k}$ by mixing the target vector $x_{i}^{k}$ with the mutated vector $v_{i}^{k}$. The crossover operation can be represented as follows:

$$U_{i,d}^{k}=\left\{ \begin{array}{ccccc} V_{i,d}^{k}, & {\text{ if randu}}_{\text{i,j}}⩽CR\;or\;j=j_{rand} & & & \\ X_{i,d}^{k}, & \text{ otherwise} \end{array}\right.$$ (12)

where $j=j_{rand}\in [1,D]$ is a random integer, *randu*_*i*,*j*_ (j∈[1,D]) is a uniformly distributed random number in (0,1). *CR* represents the crossover rate and it can set different values as needed.

### 3.3 Selection

After crossover, the selection operator is used to select the vector with a better fitness to enter the next generation. In a minimization optimization problem, the selection operator is defined as follows:

$$X_{i}^{k+1}=\left\{ \begin{array}{cc} U_{i}^{k}, & {\text{ if \$ f(U\_\{i}}^{k})⩽f(X_{i}^{k})\$\}\\ X_{i}^{k}, & \text{ otherwise}. \end{array}\right.$$ (13)

where *f*(*x*) is the objective function to be minimized. The main procedures of DE are shown in Algorithm 1.

**Algorithm 1.**

1: Initialization the population P and set some control parameters(NP,F,CR);

2: *k* = 0;

3: Evaluate the fitness value for all individuals in P;

4: **While** the terminal criterion is not satisfied **do**

5: For i=1 to NP do

6: Perform DE/rand/1 mutation operator;

7: Perform crossover operator;

8: Perform selection operator;

9: *k* = *k* + 1;

10: **end For**

11: **end while**

## 4 Differential evolution with classified mutation

Based on the objective function of these models, the parameter extraction problem can be viewed as a low-dimensional multimodal optimization problem. Some differential evolution algorithms often use fewer mutation operations to extract parameters. Since different PV models have different objective functions, the graphs of these functions in the search space are also different. It can be effective to adopt different mutation strategies in the algorithm. Here DECM achieves better parameter extraction results.

### 4.1 Different mutation strategies

In DECM, each sub-swarm has a local optimum, referred to as *X*_*lbest*_. To enhance the local exploitation capability of the entire population, it is essential to perform a local exploitation operation on high-quality individuals (*X*_*lbest*_), thereby obtaining solutions of higher quality. Employing the best individual (*X*_*best*_) enables the identification of a suitable direction, thereby decreasing the search space and improving exploration efficiency [42]. Based on the above analysis and the characteristic of multi-swarm, the local optimum (*X*_*lbest*_) adopts the following two mutation strategies.

$$V_{i,d}=X_{lbest,d}+rand\cdot (X_{best,d}-X_{r6,d})$$ (14)

$$V_{i,d}=X_{lbest,d}+rand\cdot (X_{best,d}-X_{lbest,d})+rand\cdot (X_{r7,d}-X_{r8,d})$$ (15)

where *r*6, *r*7, *r*8 are random integers in [1,*NP*], r6≠i and *r*7 ≠ 8 ≠. *rand* is a random number in the range [0,1].

In DECM, adopting the DE/rand/1 strategy for low-quality individuals in each sub-swarm can enhance the algorithm’s exploration capability [43].

### 4.2 Basic frame of DECM

Algorithm 2 shows the basic execution process of DECM. It can be seen from Algorithm 2 that DECM does not add other parameters. Compared with DE, DECM has three mutation

**Algorithm 2.**

1. Set NP, m and snum;

2. Randomly initialize the population, evaluate each individual;

3. snum sub-swarms were divided according to the index of individuals;

4. Set the local optimal individual (*X*_*lbest*_) of each sub-swarm and the best individual (*X*_*best*_) of the population;

5: **While** *FES*<*FES*_*max*_ **do**

6:   **For** i=1 **to** NP **do**

7:    **if** *X*_*i*_ is *X*_*lbest*_ of the corresponding sub-swarm

8:    **then**

9:   Use Eq 14 to perform mutation strategy;

10:    Perform selection operation;

11:    FES=FES+1;

12:    Based on the fitness value, update *X*_*lbest*_ and *f*(*X*_*lbest*_) of the corresponding sub-swarm, *X*_*best*_ and *f*(*X*_*best*_);

13:    Use Eq 15 to perform mutation strategy;

14:    Perform selection operation;

15:    FES=FES+1;

16:    Based on the fitness value, update *X*_*lbest*_ and *f*(*X*_*lbest*_) of the corresponding sub-swarm, *X*_*best*_ and *f*(*X*_*best*_);

17:     **else**

18:    Use Eq 6 to perform mutation strategy;

19:    Perform selection operation;

20:    FES=FES+1;

21:    Based on the fitness value, update *X*_*lbest*_ and *f*(*X*_*lbest*_) of the corresponding sub-swarm, *X*_*best*_ and *f*(*X*_*best*_);

22:    **end if**

23:   **end For**

24: **end While**

operations. In DECM, crossover operation is not performed which is equivalent to setting the value of parameter *CR* to 1. The multi-swarm technique is used in DECM. In Algorithm 2, *m* represents the number of individuals in each sub-swarm and *snum* represents the number of subswarms.

Here, *FES* is the number of evaluations. According to the research results of reference [44], *m*=3 is set. *NP*=60 is set in DECM.

### 4.3 The time computational complexity of DECM

The time computational complexity of DECM depends on the initialization, the evaluation of fitness functions (*FE*), the mutation operation, the selection operation, the population size (*NP*), the dimensionality of the problem (*D*). The maximum number of iterations (*G*_*max*_). The overall computational complexity of DECM can be presented by


O(DECM)=O(initialization)+O(function evaluation)+O(mutation)+O(selection)

$$=NP*(D+G_{max}(FE+D+1))$$

## 5 Experimental results and comparison analysis

To evaluate DECM’s performance, several models are used in this section. The information and data for several different photovoltaic models can be obtained from [20,45,46]. Firstly, this section gives the value range of photovoltaic model parameters. Secondly, extensive experiments are done on different PV models. Finally, the role of these different DE variants are investigated in detail and the revelent analyses are conducted. Table 3 briefly lists the operating environment of the experiment.

**Table 3:** Experimental environment.

CPU	Operating System	RAM	Software
Intel(R) i7-8700 3.20GHz	WIN 10	8.00 GB	MATLAB R2014b

The optimization range of all model parameters is the same as that of some other literatures [20]. PV module model includes Photowatt-PWP201(PVMM1), STM6-40/36(PVMM2) and STP6-120/36(PVMM3). Table 4 presents the search ranges of these parameters. The several algorithms used for comparison are DE, CODE [47], RcrIJADE [48], LSHADE [49], SHADE [50], EJAYA [5]. The parameter configurations of these algorithms are also taken from the relevant literature. In this experiment, other DE variants were configured with a population size (*NP*) of 50. For each PV problem, all the selected algorithm run 30 times independently and the maximum number of evaluations (*FES*_*max*_) is set to 50000. In order to verify the difference between DECM and other algorithms, Friedman test at the 0.05 significance level is done. The symbols ’+’, ≈ and ’-’ represent that the results of DECM are better than, similar to, and worse than the results of other algorithms, respectively.

**Table 4:** The search range of each parameter.

Parameter	SDM/DDM/TDM		PVMM1		PVMM2		PVMM3	
	LB	UB	LB	UB	LB	UB	LB	UB
*I*_*ph*_(A)	0	1	0	2	0	2	0	2
$I_{sd},I_{sd1},I_{sd2},I_{sd3}$(*μ*A)	0	1	0	50	0	50	0	50
$n,n_{1},n_{2},n_{3}$	1	2	1	50	1	60	1	50
*R*_*s*_(Ω)	0	0.5	0	2	0	0.36	0	0.36
*R*_*sh*_(Ω)	0	100	0	2000	0	1000	0	1500

### 5.1 Results on SDM, DDM and TDM

Table 5 lists the parameter extraction results of several algorithms. Based on Table 5, all algorithms can achieve the same RMSE on SDM during 30 runs. In addition, all algorithms obtain the same parameter extraction results with the same RMSE value. From Table 5, it can be clearly seen that all algorithms can achieve the same results. Compared with parameter extraction of SDM, DDM is a more serious multimodal problem. This means that more computational effort is required. As can be seen from Table 6, DECM, SHADE, LSHADE, EJAYA and RcrIJADE can achieve the better RMSE. DECM, EJAYA and RcrIJADE obtain the same parameter extraction results. Among these methods, the worst RMSE value was determined with CoDE. Table 7 presents the optimal parameter extraction results obtained from 30 runs of all algorithms on TDM. Three algorithms (EJAYA, LASHADE and RcrIJADE) achieved results that were slightly better than those obtained by the DECM. Based on the results from these three tables, it can be concluded that DECM performs relatively well in extracting parameters for the three models.

**Table 5:** Parameters estimated for SDM.

Algorithm	$I_{ph}$ (A)	$I_{sd}(\mu A)$	*n*	$R_{s}(\Omega )$	$R_{sh}(\Omega )$	RMSE
DECM	0.76078	0.32302	1.4812	0.036377	53.7185	0.00098602
SHADE	0.76078	0.32302	1.4812	0.036377	53.7185	0.00098602
LSHADE	0.76078	0.32302	1.4812	0.036377	53.7185	0.00098602
RcrIJADE	0.76078	0.32302	1.4812	0.036377	53.7185	0.00098602
CoDE	0.76078	0.32302	1.4812	0.036377	53.7185	0.00098602
DE	0.76078	0.32302	1.4812	0.036377	53.7185	0.00098602
EJAYA	0.76078	0.32302	1.4812	0.036377	53.7185	0.00098602

**Table 6:** Parameters estimated for DDM.

Algorithm	$I_{ph}$ (A)	$I_{sd1}(\mu A)$	$I_{sd2}(\mu A)$	$n_{1}$	$n_{2}$	$R_{s}(\Omega )$	$R_{sh}(\Omega )$	RMSE
DECM	0.76078	0.22597	0.74935	1.451	2	0.03674	55.4854	0.00098248
SHADE	0.76078	0.74935	0.22597	2	1.451	0.03674	55.4854	0.00098248
LSHADE	0.76078	0.74935	0.22597	2	1.451	0.03674	55.4854	0.00098248
RcrIJADE	0.76078	0.22597	0.74935	1.451	2	0.03674	55.4854	0.00098248
CoDE	0.7606	0.34574	0.06883	1.4891	1.8734	0.035959	57.9022	0.0010133
DE	0.76078	0.23307	0.68998	1.4536	2	0.036706	55.3285	0.00098251
EJAYA	0.76078	0.22597	0.74935	1.451	2	0.03674	55.4854	0.00098248

**Table 7:** Parameters estimated for TDM.

Algorithm	$I_{ph}$ (A)	$I_{sd1}(\mu A)$	$I_{sd2}(\mu A)$	$I_{sd3}(\mu A)$	$n_{1}$	$n_{2}$	$n_{3}$	$R_{s}(\Omega )$	$R_{sh}(\Omega )$	RMSE
DECM	0.76078	0.036748	7.6268e-01	7.6268e-01	1.4516	2	2	55.5209	2.1069e-07	0.0009825
SHADE	0.76078	0.036739	7.4676e-01	7.4676e-01	1.4471	2	2	55.4772	3.8321e-09	0.00098249
LSHADE	0.76078	0.03674	7.4935e-01	7.4935e-01	1.451	2	2	55.4854	2.2597e-07	0.00098248
RcrIJADE	0.76078	0.03674	6.5311e-01	6.5311e-01	2	1.4511	1.4511	55.4854	7.4935e-07	0.00098248
CoDE	0.76016	0.036922	8.4415e-01	8.4415e-01	1.8716	1.8375	1.8375	63.213	3.0706e-07	0.0011159
DE	0.76078	0.036676	6.2683e-01	6.2683e-01	1.4557	1.9981	1.9981	55.2326	2.3882e-07	0.00098261
EJAYA	0.76078	0.03674	4.8187e-9	4.8187e-9	2	1.7587	1.7587	55.4855	7.4934e-07	0.00098248

**Fig 5:**
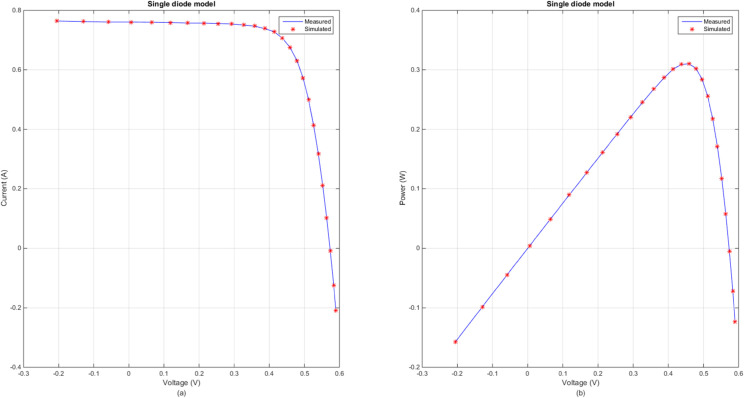
Comparison between the measured and simulated data obtained by DECM for SDM. (a) *I*–*V*, (b) *P*–*V*.

**Fig 6:**
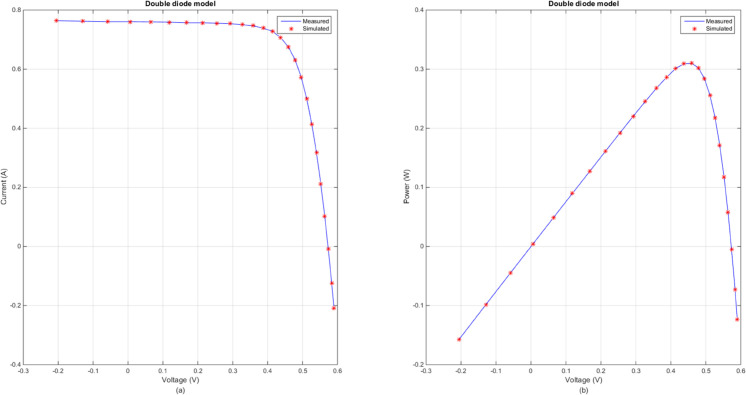
Comparison between the measured and simulated data obtained by DECM for DDM. (a) *I*–*V*, (b) *P*–*V*.

Fig 5 illustrates the comparison between the measured and simulated *I*–*V* and *P*–*V* curves for SDM using the DECM algorithm. Fig 6 illustrates the comparison between the measured and simulated *I*–*V* and *P*–*V* curves for DDM using the DECM algorithm. In both figures, the measured data are represented by solid lines, while the simulated data are depicted by dashed lines. The close alignment between the measured and simulated curves in Fig 5, highlights the high accuracy of DECM in parameter extraction for the SDM. Similarly, Fig 6 demonstrates DECM’s effectiveness in handling more complex models. These results underscore DECM’s capability to achieve high precision in fitting experimental data across different photovoltaic models.

### 5.2 Comparison on the photovoltaic modules

**Fig 7:**
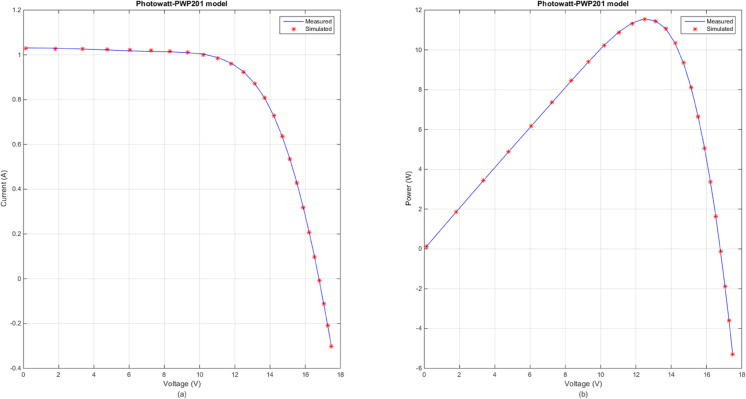
Comparison between the measured and simulated data obtained by DECM for PVMM1. (a) *I*–*V*, (b) *P*–*V*.

**Fig 8:**
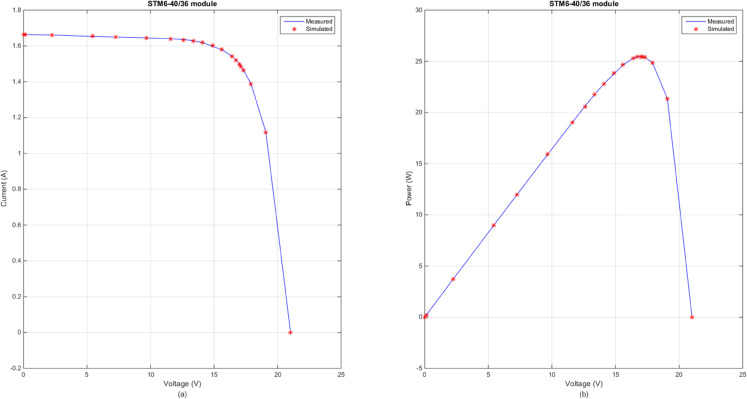
Comparison between the measured and simulated data obtained by DECM for PVMM2. (a) *I*–*V*, (b) *P*–*V*.

**Fig 9:**
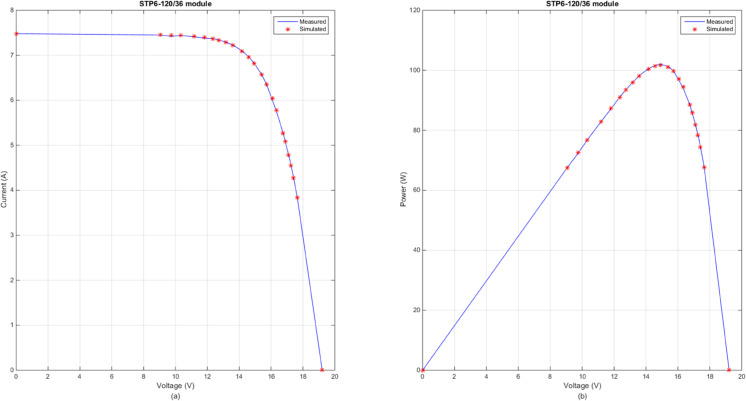
Comparison between the measured and simulated data obtained by DECM for PVMM3. (a) *I*–*V*, (b) *P*–*V*.

In this section, Table 7 lists the optimal RMSE values and parameter values under the optimal RMSE values of three photovoltaic modules obtained by the several advanced DE variants. By observing the results in Table 7, the obvious conclusion is that DECM, SHADE, LSHADE, RcrIJADE, EJAYA and DE obtained the better result on all photovoltaic modules problem. Moreover, these algorithms get the same parameter extraction results. According to Table 7, CoDE has the worst performances on PVMM2 and PVMM3. Fig 7 presents the comparison between the measured and simulated *I*–*V* and *P*–*V* curves for PVMM1, using the DECM algorithm. Fig 8 presents the comparison between the measured and simulated *I*–*V* and *P*–*V* curves for PVMM2, using the DECM algorithm. Fig 9 presents the comparison between the measured and simulated *I*–*V* and *P*–*V* curves for PVMM3, using the DECM algorithm. In these figures, the measured data are shown as solid lines, and the simulated data are shown as dashed lines. The consistent match between the measured and simulated curves across these models demonstrates the robustness and accuracy of DECM in parameter extraction for different PV module configurations.

### 5.3 Results analysis and convergence speed

**Fig 10:**
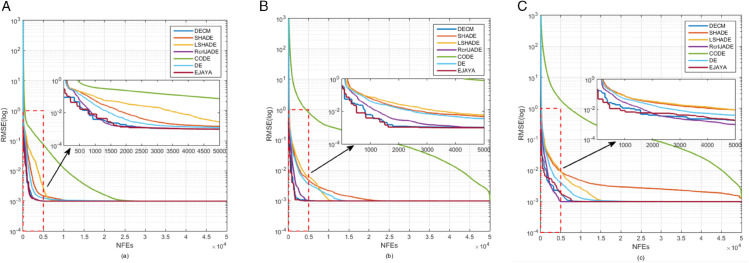
Convergence processes curves; (a) SDM, (b) DDM, (c) TDM.

**Fig 11:**
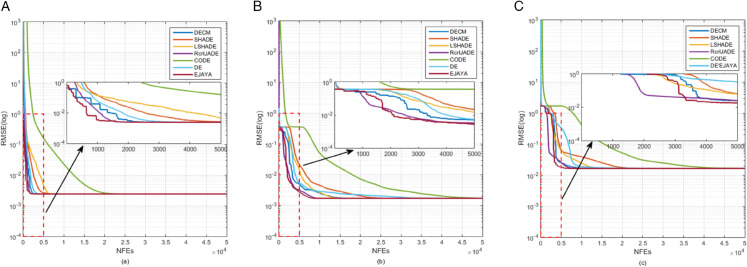
Convergence processes curves; (a) PVMM1, (b) PVMM2, (c) PVMM3.

Fig 10 presents the convergence curves for three PV models: SDM, DDM, and TDM. Fig 11 shows the convergence curves for three PV module models: PVMM1, PVMM2, and PVMM3. In both figures, the x-axis represents the number of iterations, and the y-axis represents the objective function value, the RMSE. These curves illustrate how the DECM algorithm and other algorithms converge to the optimal solution over iterations. In Fig 10, DECM’s convergence curves are compared with those of several popular algorithms for each model. DECM shows faster convergence and lower RMSE than most other algorithms. Fig 11 confirms DECM’s superior convergence again. The curves indicate that DECM can quickly approach the optimal solution in fewer iterations, highlighting its high efficiency and effectiveness in parameter extraction across different PV models. These figures clearly compare DECM’s convergence performance with that of other algorithms, emphasizing its advantages in speed and accuracy.

From Table 5 to Table 8, the optimization results of each algorithm for the model parameters were displayed. For these models, some algorithms can get better results. However, it should be emphasized that these algorithms are essentially random search algorithms. These algorithms can not guarantee to obtain better results in every run. Tables 9 and 10 record the maximum (Max), minimum (Min), average (Mean) and standard deviation (SD) of RMSE obtained by several algorithms on several PV models. The average values of RMSE in Tables 9 and 10 can well reflect the average accuracy of several methods. In Tables 9 and 10, SD can better quantify the robustness of several DE methods. Statistical results allow some conclusions to be drawn. DECM can get the best results in term of SD on SDM, PVMM1 and PVMM3. RcrIJADE obtains the best average of RMSE values on DDM. SHADE shows the best standard deviation (SD) of RMSE for PVMM2. For the double diode model, three DE algorithms (DECM, RcrIJADE, DE) get good results. On PVMM1, all algorithm get ideal results. On PVMM2, DECM, SHADE, LSHADE, EJAYA and RcrIJADE can get ideal results. On PVMM3, DECM, SHADE, EJAYA and RcrIJADE get ideal results.

**Table 8:** Comparison among different DE algorithms on PV modules.

Model	Algorithm	$I_{ph}$ (A)	$I_{sd}(\mu A)$	*n*	$R_{s}(\Omega )$	$R_{sh}(\Omega )$	RMSE
PVMM1	DECM	1.0305	3.4823	48.6428	1.2013	981.9823	0.0024251
	SHADE	1.0305	3.4823	48.6428	1.2013	981.9823	0.0024251
	LSHADE	1.0305	3.4823	48.6428	1.2013	981.9822	0.0024251
	RcrIJADE	1.0305	3.4823	48.6428	1.2013	981.9824	0.0024251
	CoDE	1.0305	3.4823	48.6428	1.2013	981.9824	0.0024251
	DE	1.0305	3.4823	48.6428	1.2013	981.9822	0.0024251
	EJAYA	1.0305	3.4823	48.6428	1.2013	981.9822	0.0024251
PVMM2	DECM	1.6639	1.7387	1.5203	0.0042738	15.9283	0.0017298
	SHADE	1.6639	1.7387	1.5203	0.0042738	15.9283	0.0017298
	LSHADE	1.6639	1.7387	1.5203	0.0042738	15.9283	0.0017298
	RcrIJADE	1.6639	1.7387	1.5203	0.0042738	15.9283	0.0017298
	CoDE	1.6639	1.7644	1.5219	0.0042214	15.9873	0.0017302
	DE	1.6639	1.7387	1.5203	0.0042737	15.9284	0.0017298
	EJAYA	1.6639	1.7387	1.5203	0.0042738	15.9283	0.0017298
PVMM3	DECM	7.4725	2.335	1.2601	0.0045946	22.2199	0.016601
	SHADE	7.4725	2.335	1.2601	0.0045946	22.2199	0.016601
	LSHADE	7.4725	2.335	1.2601	0.0045946	22.2199	0.016601
	RcrIJADE	7.4725	2.335	1.2601	0.0045946	22.2199	0.016601
	CoDE	7.4669	2.4073	1.2627	0.0045821	35.3155	0.016645
	DE	7.4725	2.335	1.2601	0.0045946	22.2199	0.016601
	EJAYA	7.4725	2.335	1.2601	0.0045946	22.2199	0.016601

**Table 9:** Statistical results of different DE algorithms for SDM, DDM and TDM.

Model	Algorithm	RMSE				±/≈	Time(s)
		Best	Mean	Worst	Std		
SDM	DECM	0.00098602	0.00098602	0.00098602	1.967e-17		41.42
	SHADE	0.00098602	0.00098602	0.00098602	2.3135e-17	+	104.03
	LSHADE	0.00098602	0.00098602	0.00098602	2.0084e-17	+	105.18
	RcrIJADE	0.00098602	0.00098602	0.00098602	3.0227e-17	≈	151.60
	CoDE	0.00098602	0.00098602	0.00098602	3.5885e-17	+	42.68
	DE	0.00098602	0.00098694	0.00099706	2.6848e-06	≈	48.08
	EJAYA	0.00098602	0.00098602	0.00098602	3.8733e-17	+	78.73
DDM	DECM	0.00098248	0.00098472	0.00098681	1.5089e-06		49.81
	SHADE	0.00098248	0.0011291	0.001857	0.00023062	+	107.38
	LSHADE	0.00098248	0.0010069	0.0016974	0.00013046	+	112.88
	RcrIJADE	0.00098248	0.00098343	0.00098602	1.5909e-06	≈	159.07
	CoDE	0.0010133	0.0012375	0.0015971	0.00013477	+	47.15
	DE	0.00098251	0.0009848	0.00098604	1.1253e-06	≈	47.77
	EJAYA	0.00098248	0.00098365	0.00098602	1.5854e-06	≈	76.52
TMD	DECM	0.0009825	0.00098581	0.0010127	5.4388e-06		1169.74
	SHADE	0.00098249	0.0014152	0.0020675	0.00035522	+	1238.73
	LSHADE	0.00098248	0.0010868	0.0018836	0.00027397	+	1239.80
	RcrIJADE	0.00098248	0.00098274	0.00098602	8.2732e-07	–	1291.49
	CoDE	0.0011159	0.0016071	0.0020113	0.00020696	+	1161.69
	DE	0.00098261	0.00098783	0.0010373	1.1483e-05	+	1165.54
	EJAYA	0.00098248	0.00098368	0.00098602	1.339e-06	–	1187.13

**Table 10:** Statistical results of different DE algorithms for PV module models.

Model	Algorithm	RMSE				±/≈	Time(s)
		Best	Mean	Worst	Std		
PVMM1	DECM	0.0024251	0.0024251	0.0024251	1.6491e-17		46.34
	SHADE	0.0024251	0.0024251	0.0024251	2.1894e-17	+	105.98
	LSHADE	0.0024251	0.0024251	0.0024251	2.2852e-17	+	107.11
	RcrIJADE	0.0024251	0.0024251	0.0024251	2.4046e-17	+	143.79
	CoDE	0.0024251	0.0024251	0.0024251	2.3917e-17	+	42.17
	DE	0.0024251	0.0024251	0.0024251	1.9784e-17	+	47.54
	EJAYA	0.0024251	0.0024251	0.0024251	4.3476e-17	≈	72.42
PVMM2	DECM	0.0017298	0.0017298	0.0017298	5.0893e-18		39.43
	SHADE	0.0017298	0.0017298	0.0017298	4.7039e-18	≈	103.35
	LSHADE	0.0017298	0.0017298	0.0017298	5.5474e-18	+	106.17
	RcrIJADE	0.0017298	0.0017298	0.0017298	6.8012e-18	+	140.74
	CoDE	0.0017302	0.0017519	0.0017991	1.671e-05	+	40.98
	DE	0.0017298	0.0018145	0.0021128	9.488e-05	+	45.07
	EJAYA	0.0017298	0.0017298	0.0017298	1.1426e-17	≈	71.33
PVMM3	DECM	0.016601	0.016601	0.016601	6.2404e-17		40.95
	SHADE	0.016601	0.016601	0.016601	7.1603e-17	+	102.81
	LSHADE	0.016601	0.017379	0.017379	0.00014218	+	104.84
	RcrIJADE	0.016601	0.016601	0.016601	2.0842e-16	≈	147.75
	CoDE	0.016645	0.016761	0.016876	5.0471e-05	+	43.94
	DE	0.016601	0.017551	0.021185	0.0010141	+	45.47
	EJAYA	0.016601	0.016601	0.016601	1.1862e-16	+	71.51

Tables 8 and 9 also list the CPU time required for each algorithm to run 30 times on each model. It can be seen that DECM, CoDE and DE run faster than SHADE, LSHADE and RcrIJADE for all cases. SHADE, LSHADE and RcrIJADE all adopt parameter adaptive technology. Obviously, parameter adaptive strategy can increase the CPU running time of these algorithms. CoDE and DE achieve poor performance on some PV models. Only RcrIJADE, EJAYA and DECM have better performance and higher robustness on all PV models. The optimization result of RcrIJADE and EJAYA on TDM is slightly better than that of DECM algorithm based on Friedman test. However, RcrIJADE is the slowest algorithm in runtime. Tables 9 and 10 show that the execution time of DECM is less than that of EJAYA and RcrIJADE on six models. The results of Friedman test demonstrate that DECM are either superior to or comparable with other algorithms. In terms of runtime, there is no significant difference between DECM, CoDE, and DE. However, these three algorithms are significantly faster than the other several algorithms in terms of runtime. In summary, DECM has better stability and speed compared to other DE algorithms based on the observation of all statistical indicators.

### 5.4 Real-world applicability and potential of DECM in renewable energy

To further test the practicality of DECM, Mono-crystal SM55, which have been applied in the real world, is selected for further testing. The experimental data for this module were collected by extracting the I-V curves from the manufacturer’ datasheets, which was measured under varying conditions of irradiance (five levels) and temperature (three conditions). In this experimental analysis, a comparison was conducted with the aforementioned several algorithms, all of which have demonstrated effectiveness. The detailed parameter configurations for this photovoltaic module is provided in [23]. Tables 11 and 13 present the parameter extraction outcomes of these algorithms under various experimental conditions. In Tables 12 and 14, four quantification indicators of RMSE are presented.

**Table 11:** Parameters estimated on Mono-crystalline SM55 under different irradiance.

Temperature	Algorithm	$I_{ph}(A)$	$I_{sd}(\mu A)$	*n*	$R_{s}(\Omega )$	$R_{sh}(\Omega )$	RMSE
*G*=200	DECM	0.69151	1.4641e-07	1.3807	0.0079617	12.4503	0.00032069
	SHADE	0.69151	1.4641e-07	1.3807	0.0079617	12.4503	0.00032069
	LSHADE	0.69151	1.4641e-07	1.3807	0.0079617	12.4503	0.00032069
	RcrIJADE	0.69151	1.4641e-07	1.3807	0.0079617	12.4503	0.00032069
	CoDE	0.69121	1.932e-07	1.4054	0.0057102	12.6641	0.00036128
	DE	0.69151	1.4642e-07	1.3807	0.0079617	12.4503	0.00032069
	EJAYA	0.69151	1.4641e-07	1.3807	0.0079617	12.4503	0.00032069
*G*=400	DECM	1.3828	1.0042e-07	1.352	0.011018	11.8625	0.00070761
	SHADE	1.3828	1.0042e-07	1.352	0.011018	11.8625	0.00070761
	LSHADE	1.3828	1.0042e-07	1.352	0.011018	11.8625	0.00070761
	RcrIJADE	1.3828	1.0042e-07	1.352	0.011018	11.8625	0.00070761
	CoDE	1.381	3.1138e-07	1.4494	0.00679	13.4726	0.0015567
	DE	1.3815	2.0705e-07	1.4127	0.0083283	12.7861	0.0011456
	EJAYA	1.3828	1.0042e-07	1.352	0.011018	11.8625	0.00070761
*G*=600	DECM	2.0709	1.5551e-07	1.3875	0.0091806	12.5019	0.00082395
	SHADE	2.0709	1.5551e-07	1.3875	0.0091806	12.5019	0.00082395
	LSHADE	2.0709	1.5551e-07	1.3875	0.0091806	12.5019	0.00082395
	RcrIJADE	2.0709	1.5551e-07	1.3875	0.0091806	12.5019	0.00082395
	CoDE	2.067	5.8898e-07	1.5088	0.0064886	17.9355	0.0035161
	DE	2.0688	3.1877e-07	1.4504	3.1877e-07	0.0077992	0.0020039
	EJAYA	2.0709	1.5551e-07	1.3875	0.0091806	12.5019	0.00082395
*G*=800	DECM	2.7604	1.4395e-07	1.3811	0.0093775	12.7744	0.00066858
	SHADE	2.7604	1.4395e-07	1.3811	0.0093775	12.7744	0.00066858
	LSHADE	2.7604	1.4395e-07	1.3811	0.0093775	12.7744	0.00066858
	RcrIJADE	2.7604	1.4395e-07	1.3811	0.0093775	12.7744	0.00066858
	CoDE	2.7562	6.3986e-07	1.5131	0.006699	18.8717	0.0043264
	DE	2.7581	2.6263e-07	1.4314	0.0083349	15.1195	0.0018716
	EJAYA	2.7604	1.4395e-07	1.3811	0.0093775	12.7744	0.00066858
*G*=1000	DECM	3.4501	1.7115e-07	1.3958	0.009143	13.4417	0.0011462
	SHADE	3.4501	1.7115e-07	1.3958	0.009143	13.4417	0.0011462
	LSHADE	3.4501	1.7115e-07	1.3958	0.009143	13.4417	0.0011462
	RcrIJADE	3.4501	1.7115e-07	1.3958	0.009143	13.4417	0.0011462
	CoDE	3.439	6.1719e-07	1.51	0.0078897	59.9726	0.0067178
	DE	3.4453	3.9866e-07	1.4692	0.0083083	21.3847	0.0043051
	EJAYA	3.4501	1.7115e-07	1.3958	0.009143	13.4417	0.0011462

**Table 12:** Statistical results on Mono-crystalline SM55 under different irradiance.

Irradiance	Algorithm	RMSE				±/≈	Time(s)
		Best	Mean	Worst	Std		
*G*=200	DECM	0.00032069	0.00032069	0.00032069	2.4677e-18		44.82
	SHADE	0.00032069	0.00032069	0.00032069	2.4073e-18	≈	108.28
	LSHADE	0.00032069	0.00032069	0.00032069	5.1158e-18	+	106.65
	RcrIJADE	0.00032069	0.00032069	0.00032069	4.0064e-18	+	141.68
	CoDE	0.00036128	0.00040135	0.00043847	1.992e-05	+	48.34
	DE	0.00032069	0.00032069	0.00032069	3.3708e-05	+	49.08
	EJAYA	0.00032069	0.00032069	0.00032069	4.5472e-18	+	70.70
*G*=400	DECM	0.00070761	0.00070761	0.00070761	5.2196e-18		45.02
	SHADE	0.00070761	0.00070761	0.00070761	7.0462e-18	+	108.38
	LSHADE	0.00070761	0.00070761	0.00070761	1.6338e-17	+	107.35
	RcrIJADE	0.00070761	0.00070761	0.00070761	1.0718e-17	+	143.95
	CoDE	0.0015567	0.0017761	0.001922	0.00010058	+	47.79
	DE	0.0011456	0.0015878	0.0026497	0.00037244	+	49.17
	EJAYA	0.00070761	0.00070761	0.00070761	2.0241e-17	+	70.39
*G*=600	DECM	0.00082395	0.00082395	0.00082395	2.0306e-17		45.41
	SHADE	0.00082395	0.00082395	0.00082395	2.1839e-17	+	108.58
	LSHADE	0.00082395	0.00082395	0.00082395	2.5939e-17	+	108.41
	RcrIJADE	0.00082395	0.00082395	0.00082395	3.4387e-17	≈	152.17
	CoDE	0.0035161	0.0039512	0.0043964	0.000249	+	43.54
	DE	0.0020039	0.0031484	0.0051182	0.00073183	+	68.54
	EJAYA	0.00082395	0.00082395	0.00082395	5.8364e-17	+	71.57
*G*=800	DECM	0.00066858	0.00066858	0.00066858	3.5486e-17		50.94
	SHADE	0.00066858	0.00066858	0.00066858	3.0383e-17	≈	111.56
	LSHADE	0.00066858	0.0010359	0.0072855	0.0013069	+	110.32
	RcrIJADE	0.00066858	0.00066858	0.00066858	4.802e-17	≈	145.94
	CoDE	0.0043264	0.0048978	0.0055537	0.00030878	+	45.23
	DE	0.0018716	0.0042527	0.010318	0.001598	+	48.53
	EJAYA	0.00066858	0.00066858	0.00066858	1.1211e-16	+	82.59
*G*=1000	DECM	0.0011462	0.0011462	0.0011462	3.0613e-17		46.21
	SHADE	0.0011462	0.0011462	0.0011462	2.4067e-17	–	107.45
	LSHADE	0.0011462	0.0025911	0.013017	0.0031587	+	108.61
	RcrIJADE	0.0011462	0.0011462	0.0011462	4.8926e-17	≈	150.02
	CoDE	0.0067178	0.0076535	0.0090948	0.00044025	+	46.59
	DE	0.0043051	0.0088522	0.014627	0.0030065	+	47.19
	EJAYA	0.0011462	0.0011462	0.0011462	7.2312e-17	+	74.89

**Table 13:** Parameters estimated on Mono-crystalline SM55 under different temperature.

Temperature	Algorithm	$I_{ph}(A)$	$I_{sd}(\mu A)$	*n*	$R_{s}(\Omega )$	$R_{sh}(\Omega )$	RMSE
$\text{T}=60{\;}^{\circ }C$	DECM	3.4946	6.9095e-06	1.4051	0.0088529	13.469	0.0037804
	SHADE	3.4946	6.9095e-06	1.4051	0.0088529	13.469	0.0037804
	LSHADE	3.4946	6.9095e-06	1.4051	0.0088529	13.469	0.0037804
	RcrIJADE	3.4946	6.9095e-06	1.4051	0.0088529	13.469	0.0037804
	CoDE	3.4944	7.1876e-06	1.4094	0.0088049	13.7429	0.0037922
	DE	3.4945	6.9576e-06	1.4059	0.0088451	13.5598	0.0037807
	EJAYA	3.4946	6.9095e-06	1.4051	0.0088529	13.469	0.0037804
$\text{T}=40{\;}^{\circ }C$	DECM	3.4691	1.1451e-06	1.4178	0.0086971	14.8075	0.0037888
	SHADE	3.4691	1.1451e-06	1.4178	0.0086971	14.8075	0.0037888
	LSHADE	3.4691	1.1451e-06	1.4178	0.0086971	14.8075	0.0037888
	RcrIJADE	3.4691	1.1451e-06	1.4178	0.0086971	14.8075	0.0037888
	CoDE	3.4674	1.5406e-06	1.4465	0.0083778	18.449	0.0042117
	DE	3.4665	1.7196e-06	1.4573	0.0082466	20.1028	0.0044676
	EJAYA	3.4691	1.1451e-06	1.4178	0.0086971	14.8075	0.0037888
$\text{T}=25{\;}^{\circ }C$	DECM	3.4501	1.7115e-07	1.3958	0.009143	13.4417	0.0011462
	SHADE	3.4501	1.7115e-07	1.3958	0.009143	13.4417	0.0011462
	LSHADE	3.4501	1.7115e-07	1.3958	0.009143	13.4417	0.0011462
	RcrIJADE	3.4501	1.7115e-07	1.3958	0.009143	13.4417	0.0011462
	CoDE	3.4401	5.8103e-07	1.5044	0.0079477	46.6578	0.0064449
	DE	3.4445	4.8319e-07	1.487	0.0080892	23.8758	0.0052469
	EJAYA	3.4501	1.7115e-07	1.3958	0.009143	13.4417	0.0011462

**Table 14:** Statistical results of different temperature on Mono-crystalline SM55.

Temperature	Algorithm	RMSE				±/≈	Time(s)
		Best	Mean	Worst	Std		
$\text{T}=60{\;}^{\circ }C$	DECM	0.0037804	0.0037804	0.0037804	6.7766e-17		47.05
	SHADE	0.0037804	0.0037804	0.0037804	8.809e-17	≈	114.05
	LSHADE	0.0037804	0.0037804	0.0037804	1.3068e-16	+	127.82
	RcrIJADE	0.0037804	0.0037804	0.0037804	1.1256e-16	+	148.23
	CoDE	0.0037922	0.0038859	0.0040793	7.4074e-05	+	45.02
	DE	0.0037807	0.0040327	0.0046029	0.00024884	+	45.92
	EJAYA	0.0037804	0.0037804	0.0037804	3.4454e-16	+	72.21
$\text{T}=40{\;}^{\circ }C$	DECM	0.0037888	0.0037888	0.0037888	5.8765e-17		46.01
	SHADE	0.0037888	0.0037888	0.0037888	5.3815e-17	–	104.64
	LSHADE	0.0037888	0.0039083	0.0069387	0.00057784	+	107.71
	RcrIJADE	0.0037888	0.0037888	0.0037888	1.3404e-16	+	143.59
	CoDE	0.0042117	0.0051247	0.005947	0.00036532	+	44.90
	DE	0.0044676	0.0064296	0.012362	0.0020762	+	46.39
	EJAYA	0.0037888	0.0037888	0.0037888	1.8721e-16	+	70.65
$\text{T}=25{\;}^{\circ }C$	DECM	0.0011462	0.0011462	0.0011462	2.7641e-17		48.04
	SHADE	0.0011462	0.0011462	0.0011462	3.5979e-17	≈	105.18
	LSHADE	0.0011462	0.0028394	0.013042	0.0030548	+	108.86
	RcrIJADE	0.0011462	0.0011462	0.0011462	5.2282e-17	+	141.77
	CoDE	0.0064449	0.0075394	0.0082047	0.00045913	+	43.89
	DE	0.0052469	0.010813	0.023909	0.0035333	+	47.18
	EJAYA	0.0011462	0.0011462	0.0011462	7.9495e-17	+	71.80

#### 5.4.1 Results on Mono-crystalline SM55 under different irradiance.

The experimental configuration maintained constant ambient temperature at $25{\;}^{\circ }C$ while applying five distinct irradiance levels (200 W/m2, 400 W/m2, 600 W/m2, 800 W/m2, and 1000 W/m2) to monocrystalline SM55 photovoltaic modules. Table 12 presents comparative analysis of parameter estimation errors through root mean square error (RMSE) metrics across optimization algorithms. Notably, DECM demonstrates superior optimization capability by achieving minimum RMSE values of 0.00032069, 0.00070761, 0.00082395, 0.00066858, and 0.0011462 across respective irradiance intensities, outperforming some algorithms. The statistical distributions presented in Table 12 reveals significant performance advantages of the proposed DECM. Quantitative analysis demonstrates that DECM achieves superior optimization precision. It is particularly noteworthy that the standard deviation values effectively reflect the superior stability of DECM. In terms of the Friedman test, under five different irradiances, the performance of the DECM is superior to other algorithms. In terms of execution time, these three algorithms (DECM, DE, and CoDE) are significantly faster than the other four.

#### 5.4.2 Results on mono-crystalline SM55 under different temperature.

Experimental validation was conducted under standardized irradiance conditions (1000 W/m2) applied to monocrystalline SM55 photovoltaic modules across three thermal regimes ($25{\;}^{\circ }C$,$40{\;}^{\circ }C$,$60{\;}^{\circ }C$). As quantitatively demonstrated in Table 14, the proposed DECM algorithm achieves superior optimization performance with minimum RMSE metrics of 0.0011462 ($25{\;}^{\circ }C$), 0.0037888 ($40{\;}^{\circ }C$), and 0.0037804 ($60{\;}^{\circ }C$), respectively. In terms of the RMSE metric, Table 14 indicates that the four algorithms (DECM, SHADE, RcrIJADE, and EJAYA) achieved comparable and satisfactory results. Among these four algorithms, the DECM algorithm exhibits the shortest runtime. Overall, considering the results of the Friedman test and the runtime comprehensively, the DECM algorithm outperforms the other algorithms.

#### 5.4.3 Potential for other renewable energy applications.

Beyond photovoltaic systems, DECM’s optimization capabilities hold promise for other renewable energy applications. Its ability to efficiently solve complex optimization problems can be leveraged in various domains, such as wind energy and hydro energy. Other evolutionary algorithms have already found practical applications in renewable energy systems [51–54]. In wind energy systems, accurate parameter estimation is essential for optimizing turbine performance and maximizing energy capture. DECM can be applied to optimize parameters such as blade pitch angles, rotor speeds, and control strategies. By fine-tuning these parameters, DECM can help increase the efficiency of wind turbines, leading to higher energy output and reduced operational costs. For hydro energy systems, DECM can be used to optimize the design parameters of turbines, generators, and water flow control mechanisms. Accurate optimization of these parameters ensures that the hydro energy system operates at peak efficiency, maximizing energy production while minimizing environmental impact.

### 5.5 The effect of different mutation strategies

This section focuses primarily on the influences of three mutation strategies. The following method is used for this investigation. When one mutation strategy is removed and the other two mutation strategies remain unchanged, the performance of DECM is observed. A variant of DECM that removed the mutation strategy of Eq 14 in DECM is referred to as DECM1. A variant of DECM that removed the mutation strategy of Eq 15 in DECM is referred to as DECM2. A variant of DECM that removed the mutation strategy of Eq 6 in DECM is referred to as DECM3. In DECM3, the evolution of each individual mainly depends on Eqs 14 and 15. SDM and DDM are selected as test models. The results obtained are shown in Table 15. It can be clearly seen that DECM and DECM2 can achieve the better results in SDM. As can be seen from Table 15, compared to the other three algorithms, DECM has the best performance on DDM according to four statistical indicators of RMSE. Due to the combination of three different mutation strategies, DECM performs better than the other three algorithms for SDM and DDM. Obviously, these three differential strategies play an important role in DECM. In short, any mutation strategy can improve the performance of DECM. Based on the above discussion, it can be concluded that these mutation strategies used in DECM are reasonable and effective.

**Table 15:** Statistical results of several DECM variants for two models.

Model	Algorithm	RMSE			
		Best	Mean	Worst	Std
SDM	DECM	0.00098602	0.00098602	0.00098602	1.967e-17
	DECM1	0.00098602	0.00098602	0.00098602	1.8982e-17
	DECM2	0.00098602	0.0054084	0.0049133	0.01173
	DECM3	0.00098602	0.0011052	0.0045626	0.000653
DDM	DECM	0.00098248	0.00098472	0.00098681	1.5089e-06
	DECM1	0.00098265	0.00099258	0.0011954	3.8333e-05
	DECM2	0.00098248	0.004735	0.046317	0.010028
	DECM3	0.00098248	0.001056	0.0024307	0.00027307

### 5.6 Analyzing the trade-off between computational time and accuracy

In the field of optimization algorithms, achieving a balance between computational efficiency and solution accuracy is crucial. Table 16 compares DECM with other algorithms across three models, revealing the trade-off relationships among them. The contents of the 3rd, 4th, 6th, and 7th columns of Table 16 are derived from Table 9. DECM consistently demonstrates superior or competitive performance in terms of the average change rate, indicating its robustness in maintaining or improving the accuracy of parameter extraction. For example, in the SDM, DECM achieves an average change rate of -0.093%, outperforming DE. This result implies that the error has been reduced, and thus the (prediction) accuracy has been improved. When examining computational time, DECM also exhibits favorable trade-offs. The time change rate data shows that DECM can match or even significantly surpass other algorithms in execution speed. For instance, in the DDM, DECM shows a +4.26% change rate relative to DE. This is particularly noteworthy because the faster computation speed does not come at the cost of sacrificing the accuracy of extracted parameters. This balance is even more pronounced in complex models like the TDM, where DECM not only maintains high accuracy but also achieves comparable or better time efficiency than other algorithms.As demonstrated in the comparative analysis of Table 16, the DECM algorithm exhibits compelling effectiveness in achieving this balance, especially when applied to PV model parameter extraction.In summary, DECM serves as a robust optimization tool that effectively manages the trade-off between computational time and solution accuracy.

**Table 16:** Change rates of DECM vs. other algorithms on two metrics.

Model	Algorithm	DECM Mean	Other Alg. Mean	Change Rate	DECM Time (s)	Other Alg. Time (s)	Change Rate
SDM	DE	0.00098602	0.00098694	-0.093%	41.42	48.08	-13.86%
	SHADE	0.00098602	0.00098602	0%	41.42	104.03	-60.18%
	LSHADE	0.00098602	0.00098602	0%	41.42	105.18	-60.62%
	RcrIJADE	0.00098602	0.00098602	0%	41.42	151.60	-72.67%
	EJAYA	0.00098602	0.00098602	0%	41.42	78.73	-47.38%
DDM	DE	0.00098472	0.0009848	-0.008%	49.81	47.77	+4.26%
	SHADE	0.00098472	0.0011291	-12.78%	49.81	107.38	-53.61%
	LSHADE	0.00098472	0.0010069	-2.20%	49.81	112.88	-55.87%
	RcrIJADE	0.00098472	0.0009834	+0.131%	49.81	159.07	-68.68%
	CoDE	0.00098472	0.0012375	-20.42%	49.81	47.15	+5.64%
	EJAYA	0.00098472	0.0009365	+0.109%	49.81	76.52	-34.90%
TDM	DE	0.00098581	0.0009878	-0.204%	1169.74	1165.54	+0.36%
	SHADE	0.00098581	0.0014152	-30.34%	1169.74	1238.73	-5.57%
	LSHADE	0.00098581	0.0010868	-9.29%	1169.74	1239.80	-5.65%
	RcrIJADE	0.00098581	0.0009827	+0.312%	1169.74	1291.49	-9.42%
	CoDE	0.00098581	0.0016071	-38.65%	1169.74	1161.69	+0.69%
	EJAYA	0.00098581	0.0009368	+0.217%	1169.74	1187.13	-1.46%

### 5.7 Discussions

Based on the above experimental results and relevant analysis, the following insights can be obtained:

(1) Considering both the execution time and the results of the Friedman test, DECM can be regarded as an excellent algorithm for parameter extraction from PV models compared with other algorithms.

(2) Although some DE algorithms (SHADE, LSHADE, RcrIJADE) can produce better results using parameter adjustment technology, these algorithms also consume more running time. Other algorithms that do not use an adaptive strategy have a shorter algorithm running time. Therefore, the parameter adjustment strategy can be a main reason for extending the algorithm running time. It is necessary to design the appropriate techniques to shorten the running time. The computational speed of an algorithm can affect the scope of the algorithm.

(3) DECM employs three different mutation strategies. This indicates that the combination of different mutation strategies can effectively solve the problem of parameter extraction in photovoltaic models. Using different mutation strategies does not increase the running time of the algorithm.

## 6 Conclusion

The DECM algorithm proposed in this paper represents a significant advancement in the field of parameter extraction for photovoltaic models. By adopting a multi-swarm strategy and role-based classification mutation strategy, DECM effectively avoids crossover operations and parameter tuning, resulting in enhanced simplicity and operational efficiency. Compared with existing studies, DECM not only achieves more accurate parameter extraction results but also demonstrates higher robustness and faster convergence speed. The simplicity and efficiency of DECM make it a promising tool for practical applications in photovoltaic systems. The some findings can be summarized as follows.

(1) It is a very important and promising research direction to shorten the time for computing the algorithm.

(2) It is necessary to analyze the role of different mutation strategies in parameter extraction. To design powerful parameter extraction algorithms, further combining these mutation strategies can be an effective approach.

(3) In terms of improving the performance of algorithms, developing new mutation strategies can be a promising and effective method. It is also a worthwhile research direction to integrate the mutation strategy into other metaheuristic algorithms. Some external environmental factors have an important influence on the current and voltage of the PV system. There is an urgent need to design new models taking these environmental factors into account.

In the future, the following works will be devoted to the design of new mutation strategies, the study of the role of the combination of these mutation strategies in parameter extraction, and the design of more advanced DE variants. In addition, these DE variants are used to solve other practical problems in photovoltaic systems.
